# Dataset supporting the proteomic characterization of human corneal epithelial cells with HSV-1 infection

**DOI:** 10.1016/j.dib.2019.104579

**Published:** 2019-09-28

**Authors:** Zhi-Yi Xu, Jia-Hui Li, Mei-Jun Li, Wen-Lin Zheng, Hong-Wei Pan

**Affiliations:** a Department of Ophthalmology, The First Affiliated Hospital, Jinan University, Guangzhou, China; b Institute of Ophthalmology, School of Medicine, Jinan University, Guangzhou, China; c Department of Public Health and Preventive Medicine, Jinan University, Guangzhou, China

**Keywords:** HSV-1, Corneal epithelial cell, Proteomics, iTRAQ, MRM, Bioinformatic analysis

## Abstract

HSV-1 infection in cornea can cause corneal ulcer, scar formation and neovascularization, and finally lead to severe visual impairment. The corneal epithelium is the first barrier against HSV-1 infection, but the host-virus interaction in human corneal epithelial cells (HCECs) in the process is still not well understood. We applied iTRAQ based proteomic approach to investigate the dynamic change of the protein expression profile in HCECs with a view to gain insight into the host response to HSV-1 infection. Bioinformatic analysis of these dysregulated proteins help us to find the potential gene function and signaling pathway with which these dysregulated proteins are associated. In this work, we present the supporting information for the proteomic characterization for better share and reuse. The main methodological approaches and major findings of the proteomic experiments are described in [1].

**Table:** Specifications Table

Subject	*Biology*
specific subject area	*Cellular proteomics*
Type of data	*Tables and figures*
How data was acquired	*iTRAQ coupled with LS-MS/MS*,*MRM*, *bioinformatic analysis*
Data format	*Raw and analyzed data*
Parameters for data collection	*Primary human corneal epithelial cells cultured in vitro were infected or mock-infected with HSV-1*.
Description of data collection	*Cell lysates were digested*, *labeled with iTRAQ*, *analyzed with LC-MS/MS and validated by MRM*.
Data source location	*Jinan University*, *Guangzhou*, *China*
Data accessibility	*Data are provided with this article*
Related research article	Y.H. Cui, Q. Liu, Z.Y. Xu, J.H. Li, Z.X. Hu, M.J. Li, W.L. Zheng, Z.J. Li, H.W. Pan, Quantitative proteomic analysis of human corneal epithelial cells infected with HSV-1, Exp Eye Res 185 (2019) 107664. https://doi.org/10.1016/j.exer.2019.05.004

**Table:** 

**Value of the Data** • This data presents an overview of protein interaction network which can provide clues to other researchers to screen the vital proteins or pathways in immune response to HSV-1 infection. • For future investigations, this proteomic characterization can be integrated with transcriptional and metabolic analysis for multi-omic strategy which may help clarify an elusive mechanism. • This data helps to better understand the pathogenesis and explore potential target for treatment of herpes simplex keratitis.

## 1 Data

We have previously reported the global transcriptional changes in HCECs induced by HSV-1 infection, including both protein-coding RNAs and long non-coding RNAs [2]. We also performed proteomic characterization of HCECs with HSV-1 infection and reported the main findings in our recent publication [1].

**Fig. 1:**
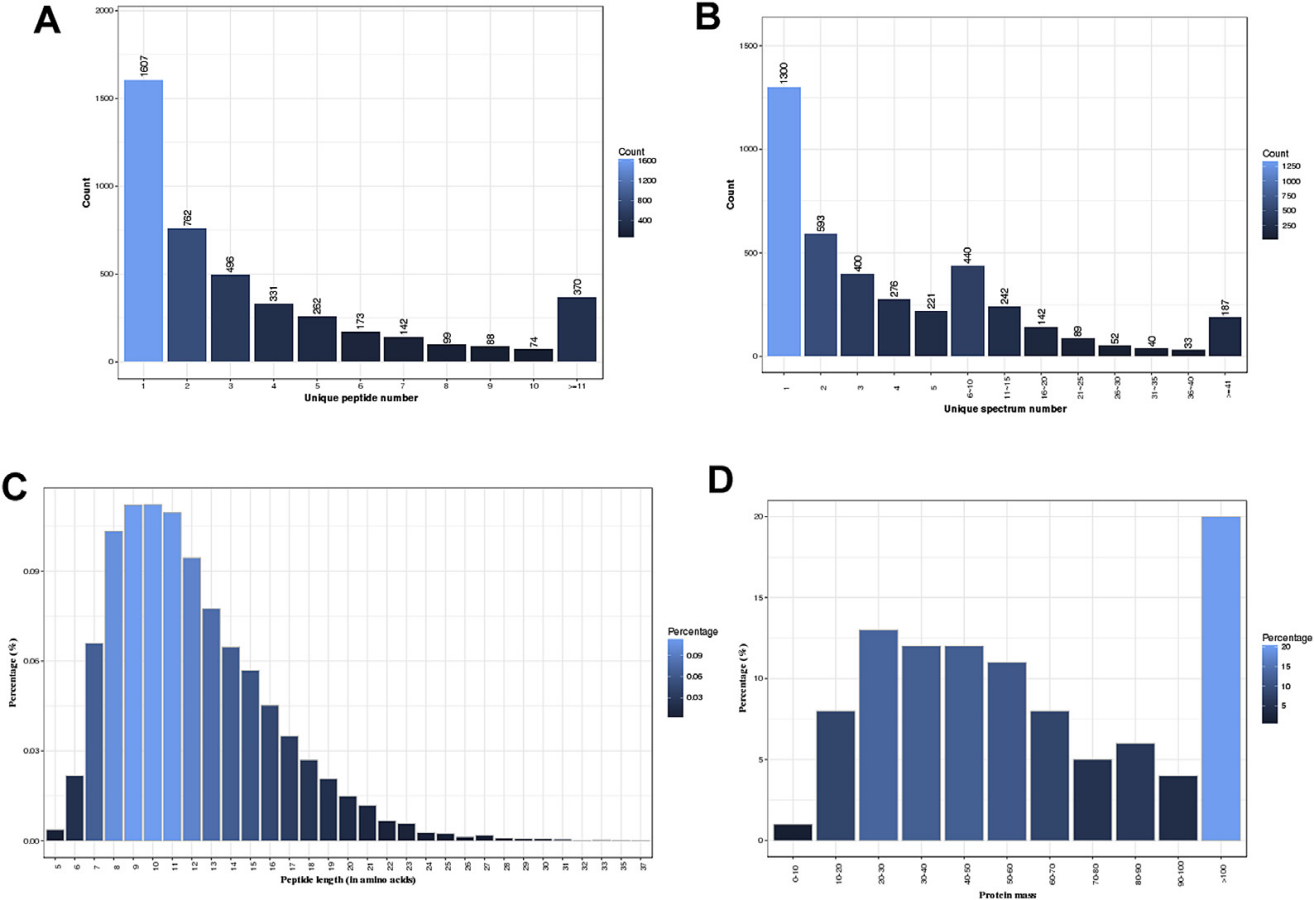
The characteristics of the proteins identification with mass spectrometry. A. Distribution of unique peptide number for individual protein. B. Distribution of unique spectrum number for individual protein. C. Distribution of peptides length. D. Distribution of proteins mass.

**Fig. 2:**
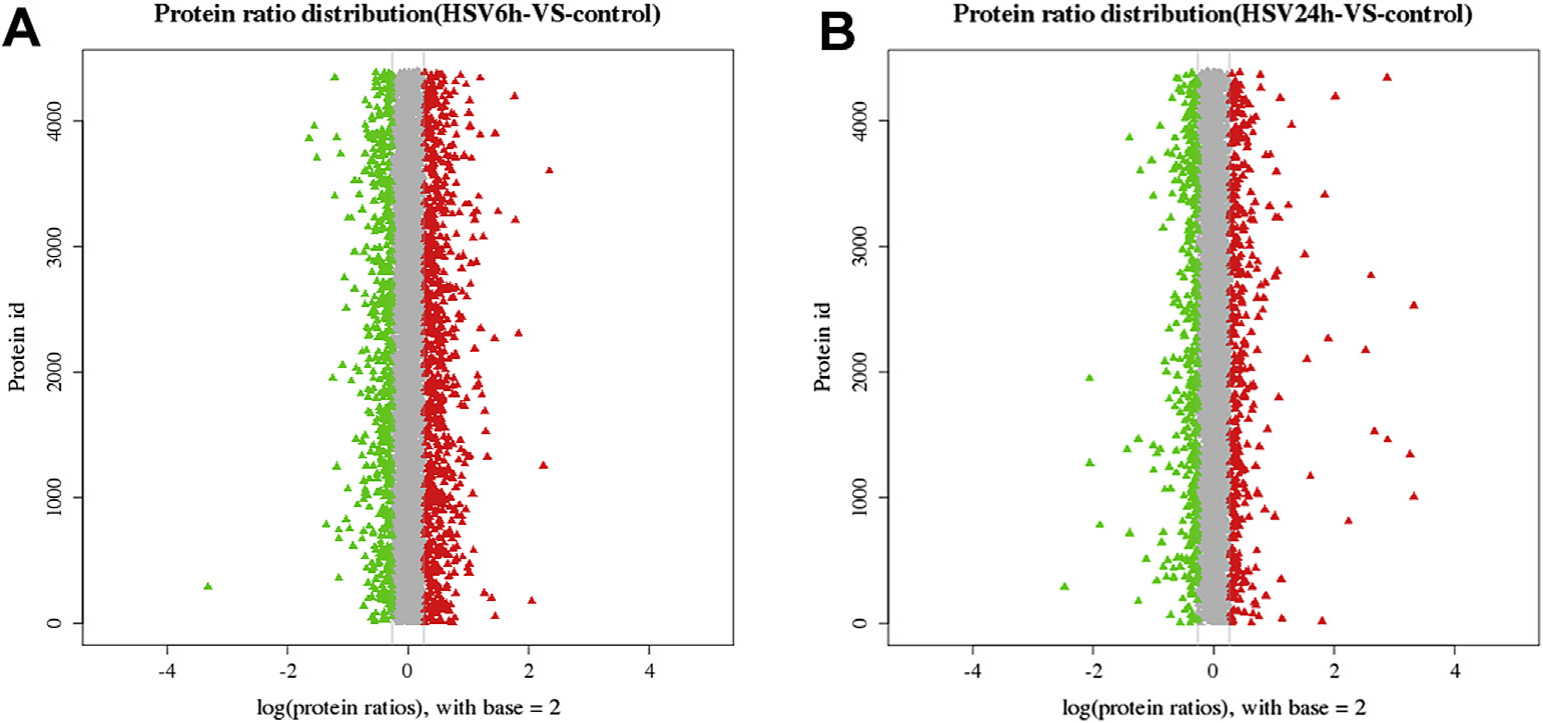
Volcano plots showing the differential expression in HCECs proteins at 6 hour post infection (hpi) (A) and 24 hpi (B). The red triangle in the plots indicates a upregulated protein while the green triangle indicates a downregulated protein with fold change >1.2 and P < 0.05 in HSV-1 infection group compared with control group.

The dataset in this article shows the proteins profiles in HCECs after HSV-1 infection both in early and late stage, as well as the detailed information from bioinformatic analysis. Fig. 1 describes the characteristics of the proteins identification with mass spectrometry. Fig. 2 describes the differential expression in HCECs proteins at 6 hour post infection (hpi) (A) and 24 hpi (B). Table 1 shows the comparison of proteins expression analyzed by iTRAQ and MRM. Supplementary Table 1 and Supplementary Table 2 show the mass spectrometry data of the top 20 dysregulated proteins at 6 hpi and 24 hpi, respectively. Supplementary Table 3 and Supplementary Table 4 show detailed list of GO terms and KEGG pathways associated with the dysregulated proteins.

**Table 1:** Comparison of proteins expression analyzed by iTRAQ and MRM.

Access number	Gene symbol	HSV-1 infection/mock at 6 hpi		HSV-1 infection/mock at 24 hpi	
		iTRAQ	MRM	iTRAQ	MRM
O00299	CLIC1	2.11	3.02	1.3	1.45
P00338	LDHA	2.29	3.41	1.3	1.23
P04406	GAPDH	2.37	4.93	1.3	1.48
P07686	HEXB	0.59	0.35	0.97	0.59
P09525	ANXA4	0.96	0.60	1.45	1.10
P09960	LTA4H	2.15	4.87	1.2	1.81
P10909	CLU	3.44	0.52	0.65	0.63
P11940	PABPC1	1.23	1.80	1.41	1.86
P14324	FDPS	2.42	9.65	1.15	1.94
P22626	HNRNPA2B1	0.87	0.49	0.68	0.29
P23284	PPIB	0.59	0.24	0.98	0.60
P26639	TARS	2.2	3.71	1.13	1.41
P27797	CALR	0.56	0.23	1.02	0.74
P29590	PML	0.7	0.51	0.52	0.29
P30040	ERP29	0.61	0.22	0.94	0.56
P30048	PRDX3	0.62	0.30	0.98	0.62
P53396	ACLY	2.22	4.50	1.2	1.45
P55060	CSE1L	2.25	2.82	1.13	1.28
P61604	HSPE1	0.59	0.19	1.08	0.70
P63244	RACK1	2.4	4.06	1.28	1.45
P78527	PRKDC	1	0.69	0.62	0.18
Q00059	TFAM	0.85	0.34	0.62	0.23
Q15056	EIF4H	0.86	0.46	2.06	2.84
Q8NC51	SERBP1	1.24	0.99	1.44	1.10
Q9P2B2	PTGFRN	1.01	1.13	0.73	0.61

## 2 Experimental design, materials, and methods

### 2.1 Cell culture and experimental design

The primary human corneal epithelial cells (HCECs) obtained from ATCC were cultured as the protocol recommended by the provider. After reaching 80%–90% confluence, the cells were inoculated with HSV-1 at the MOI of 0.1. HCECs were transferred to complete medium from the basal medium after one hour of absorption. HCECs without HSV-1 inoculation were served as controls. For all the three groups, 6 hpi infection group, 24 hpi infection and mock-infection group, we collected two replicate samples from independent experiments.

### 2.2 Protein preparation, iTRAQ labeling, LS-MS/MS and MRM

The cells cultured in flask were washed with PBS before lysed with lysis buffer. The resulting protein solution was added with 10 mM DTT. After sonication treatment followed by centrifugation, the protein solution was incubated with IAM(55 mM). The protein solution was mixed with 100mM TEAB, and then subject to digestion with trypsin Gold. The digested peptides were desalted, vacuum-dried and resuspended in 0.5M TEAB with vortexing. Peptide was labeled with iTRAQ Reagent 8-plex Kit according to the instruction of the manufacturer. with autosampler in LC-20AD nano-HPLC instrument, each fraction reconstituted in solution A was introduced into C18 trap column and then eluted with gradient solvent B. Mass spectrometry was performed on the platform of TripleTOF 5600 System.

Multiple reaction monitoring (MRM), which is developed as kind of targeted proteomic approach, is applied for an efficient tool in validation of quantitative proteomic analysis [3]. The protein samples were digested into peptides and then spiked with β-galactosidase for data normalization. QTRAP 5500 mass spectrometer equipped with LC-20AD nanoHPLC system were used as the platform for MRM analyses.

### 2.3 Data analysis and bioinformatic analysis

The raw MS data were used to generated MGF files by ProteoWizard tool msConvert, which were searched against the database of human with Mascot version 2.3.02. For confident identification of the proteins, at least one unique peptide is required. We utilized an automate software, IQuant, to quantitatively analyze the isobaric tags labeled peptides, as previously reported [4]. The characteristics of protein identification with mass spectrometry was shown in Fig. 1. We used a cutoff of 1.2-fold change to determine the differential expression of proteins. The differential expression of proteins in HCECs induced by HSV-1 infection at 6 hpi and 24 hpi was shown in Fig. 2. The mass spectrometry data of the top 20 dysregulated proteins at 6 hpi and 24 hpi were shown in Supplementary Table 1 and Supplementary Table 2, respectively.

For LC-MRM–MS, the raw file was integrated with Skyline software. The chromatography of a given peptide was determined with an iRT strategy. MSstats software was used with mixed-effects model. The adjusted P value of FDR under 0.05 was considered to be significant. We compared the relative ratios of 25 proteins in 6 hpi and 24 hpi versus mock infection obtained from iTRAQ analysis and MRM analysis, as listed in Table 1.

We carried out bioinformatic analysis of the dysregulated proteins in the proteomic characterization. With the online tools of DAVID, we performed gene ontology analysis and KEGG pathway analysis to explore the potential biological implication of these proteins. A P value < 0.05 was used as cut-off in the Fisher's exact test to determine the significant overlap on a given gene sets. The detailed list of GO terms and KEGG pathways associated with the dysregulated proteins at 6 and 24 hpi were shown in Supplementary Table 3 and Supplementary Table 4.
