# Supplementary material for: Dataset supporting the proteomic characterization of human corneal epithelial cells with HSV-1 infection
Source: Data Brief. 2019 Sep 28;27:104579. doi: 10.1016/j.dib.2019.104579 (PMC6817629; doi:10.1016/j.dib.2019.104579)
Supplement: Multimedia component 1 [file mmc1.doc]

Supplementary table 1. GO terms associated with the dysregulated proteins in HCECs in response to HSV-1 infection at 6 and 24 hpi

| **GO category** | **GO ID** | **GO term** | | **Proteins involved** | **P value** |
| --- | --- | --- | --- | --- | --- |
| **down-regulated proteins at 6 hpi** | | | | | |
| BP | GO:0000398 | mRNA splicing, via spliceosome | | Q12874, P26368, P09234, Q9BWJ5, P62304, Q07955, Q13435, P55769, P62316, Q16629, O43390, Q05519, O75400, Q15393, P08621, Q14103, Q15459, O43809, Q01130, P09661 | 5.76E-13 |
| BP | GO:0045454 | cell redox homeostasis | | P30101, Q9H299, Q8NBS9, P09622, Q13162, P13667, P30044, Q96HE7, P10599, O95881, P30048, P07237, Q9BS26 | 1.07E-11 |
| BP | GO:0006457 | protein folding | | P30101, Q9HAV7, Q8NBS9, P27797, P30533, P30405, P30040, P13667, P61604, P14314, Q14697, Q96HE7, P10599, P50502, Q96AY3, P26885, Q9BS26 | 2.40E-11 |
| BP | GO:0006397 | mRNA processing | | Q12874, P26368, Q07021, Q16630, Q07955, Q13435, Q9UHX1, Q16629, O43390, Q05519, P08621, Q15393, Q9UKA9, Q15459, O43809, Q01130 | 2.40E-10 |
| BP | GO:0006099 | tricarboxylic acid cycle | | P31040, P40926, P50213, P07954, P36957, P09622, P08559, Q9P2R7 | 9.71E-09 |
| CC | GO:0070062 | extracellular exosome | | P07355, P36405, P12429, P07686, Q15819, P10412, P11166, P17900, P61604, P18085, P54819, P50502, P13646, Q16629, P31949, P08758, P36957, P49419, P10619, P06702, P40926, P06703, Q8NBS9, P60903, Q13011, P80303, P05109, P42785, Q07955, Q13510, P26447, P06753, P47756, P62316, P10809, P04179, P10599, O75131, P07237, P67936, P19338, Q9P2R7, Q9BS26, Q16698, P27797, Q13162, O00515, P62304, P05164, P52907, P30044, Q9UBR2, P22307, P30048, P11021, P26885, Q9H299, P30101, Q9NVJ2, Q9Y2Q5, P50454, O75340, P30040, P20337, P47929, Q01469, P06576, Q9NYU2, P06396, Q14697, P12532, P23284, P30084, P07339, P07954, Q14103, Q9Y4L1, P07602, Q01130, P23381, Q14247 | 2.88E-23 |
| CC | GO:0005788 | endoplasmic reticulum lumen | | O15460, P30101, Q8NBS9, P27797, Q15293, P50454, P30040, P13667, P14314, Q9NYU2, Q14697, P23284, Q9UBR2, Q96HE7, O95881, P07237, P11021, Q96AY3, Q9Y4L1, Q8NBJ5, Q9BS26 | 1.02E-15 |
| CC | GO:0005759 | mitochondrial matrix | | P49748, P40926, Q16698, Q9HAV7, Q07021, P30405, O95571, P09622, P61604, P06576, P30044, P10809, P04179, P30084, P30048, Q96E11, P30049, P50213, P07954, O00330, P36957, P08559, Q9P2R7, P49419 | 3.25E-14 |
| CC | GO:0005739 | mitochondrion | | P49748, Q9H2W6, Q16698, Q5VTU8, Q07021, O95571, P09622, Q13162, P05164, P17900, P31040, P61604, P30044, Q9BYD6, Q8TCS8, P22307, P30048, P30049, P11021, P36957, P08559, P49419, P19404, Q12849, P40926, Q9HAV7, P14854, Q13011, P99999, P06576, P12532, P10809, P10599, P04179, P30084, Q96E11, P50213, P07954, Q14249, Q15067, P07602, Q9P2R7 | 3.19E-12 |
| CC | GO:0005681 | spliceosomal complex | | Q13435, Q12874, P62316, P26368, O43390, P08621, Q15393, Q9UKA9, Q15459, P09661, Q01130, P62304 | 9.84E-10 |
| MF | GO:0044822 | poly(A) RNA binding | | P07355, Q12905, Q12874, Q9Y3Y2, Q16630, P10412, P61604, Q16629, P07305, O43390, Q13428, O43809, P40926, Q8IWX8, Q92522, P06748, Q07955, P55145, P26447, P16403, P62316, P10809, P10599, O75131, P07237, P19338, Q15459, P09661, P11387, P27797, P13667, P55769, Q9BYD6, Q8TCS8, Q9UHX1, Q99575, Q9UKA9, P30101, Q12849, P26368, P09234, P50454, P20810, Q13435, Q96SI9, Q14697, P23284, Q05519, O75400, P08621, P78346, Q14103, Q01130 | 4.51E-22 |
| MF | GO:0003723 | RNA binding | | Q12905, Q12874, Q16630, P62304, P55769, Q9BYD6, Q8TCS8, O43390, Q9UKA9, O43809, Q12849, Q9NQT4, P26368, P78406, Q8IWX8, P06748, Q07955, Q96SI9, P14314, O75400, Q15393, P08621, P78346, Q14103, P19338, Q15459 | 1.28E-10 |
| MF | GO:0003756 | protein disulfide isomerase activity | | P13667, P30101, Q96HE7, Q8NBS9, P07237, P30040, Q9BS26 | 5.22E-08 |
| MF | GO:0051082 | unfolded protein binding | | P61604, Q9NYU2, Q9HAV7, P10809, P23284, P50502, P27797, P30533, P11021, P50454, P06748 | 1.23E-07 |
| MF | GO:0098641 | cadherin binding involved in cell-cell adhesion | | P07355, Q9UHB6, Q96C19, Q16643, Q92522, P20810, O00515, Q9NYL9, P52907, Q9UHX1, P47756, P31949, P11021, Q14247 | 5.61E-06 |
| **Up-regulated proteins at 6 hpi** | | | | | |
| BP | GO:0006413 | | translational initiation | P60228, P36578, P62277, P62753, P27635, P61353, P32969, P62280, P18621, P62269, P61247, O00303, P62263, P23396, P62851, P15880, Q02543, P49207, P39019, O75821, P62910, P62913, P60842, P20042, Q13347, P11940, Q9BY44, P61254, O60841, P56537, P46776, P46778, P35268, P46779, P61221, P62249, P84098, P62906, P62241, P62244, P46782, P46781, P60866, P05388, Q14152, P62081, P83731, P25398, P55884, Q9Y262, P62841, P50914, P18077, Q99613, P40429, P26373, P41091, P62847 | 3.70E-65 |
| BP | GO:0006614 | | SRP-dependent cotranslational protein targeting to membrane | P36578, P46776, P35268, P46778, P46779, P62277, P62753, P27635, P61353, P32969, P62280, P62249, P18621, P62269, P84098, P62906, P61247, P62241, Q9UNL2, P62244, P62263, P23396, P46782, P62851, P46781, P60866, P15880, Q02543, P49207, P39019, P05388, P83731, P62081, P62910, P62913, P25398, O76094, P61254, P62841, P50914, P18077, P40429, P26373, P62847 | 2.52E-51 |
| BP | GO:0000184 | | nuclear-transcribed mRNA catabolic process, nonsense-mediated decay | P60228, P36578, P63151, P46776, P35268, P46778, P46779, P30153, P62277, P62753, P27635, P61353, P32969, P62280, P62249, P18621, P62269, P84098, P61247, P62906, P62241, P62244, P62263, P23396, P46782, P62851, P46781, P60866, P15880, Q02543, P49207, P39019, P05388, P83731, P62081, P62910, P62913, P25398, P11940, P61254, P62841, P50914, P18077, P40429, P26373, P62847 | 4.68E-49 |
| BP | GO:0019083 | | viral transcription | P36578, P46776, P35268, P46778, P46779, P62277, P62753, P27635, P61353, P32969, P62280, P62249, P18621, P62269, P84098, P61247, P62906, P62241, P62244, P62263, P23396, P46782, P62851, P46781, P60866, P15880, Q02543, P49207, P39019, P05388, P83731, P62081, P62910, P62913, P25398, P61254, P62841, P50914, P18077, P40429, P26373, P62847 | 5.13E-44 |
| BP | GO:0006412 | | translation | P36578, P46776, P14868, P35268, P46778, P46779, P62277, P62753, P27635, P61353, P32969, P62280, P62249, P18621, P62269, P84098, P61247, P62906, P62241, P62244, P62263, P23396, P46782, P62851, P46781, P60866, P15880, Q02543, P49207, P39019, P05388, P26639, Q92616, P19525, P83731, P62081, P62910, P62913, P25398, P61254, Q9NSD9, P62841, P50914, P18077, P40429, P26373, P62847 | 5.58E-34 |
| CC | GO:0005829 | | cytosol | P46940, Q13200, P36578, O60610, P78417, Q9BR76, P27635, P43034, P62753, O43175, P23526, P14324, P07737, P53621, P52789, P62269, P62263, P23396, P62851, Q04828, P49588, O00212, P41252, O60701, P54577, O75369, P20042, P28074, Q13347, P00558, Q9NSD9, Q16831, P13796, P43686, P40227, Q01813, P62837, P35606, Q9NZN4, P16152, P62249, P00491, Q9Y678, P84098, O75083, P62241, P62244, P07947, P63104, P00338, P61088, P83731, Q13085, Q9BZF3, P07814, P62841, O43592, P50914, P14618, P26373, P62847, P53618, P60228, O43242, O60443, P30153, P60709, P61353, P29401, O00303, O43747, P15880, P40925, P21333, Q04446, Q07960, O60841, Q13418, P11413, Q10567, P51665, P56192, Q96TA1, P46776, P14868, P46778, P46779, P61981, P07900, O00429, P46782, Q16401, P46781, P26639, P62081, P17096, P25398, P26640, P49368, P13639, Q9Y2A7, P62333, P18077, Q99613, Q96QK1, P04406, Q01082, Q12904, Q9Y6Q5, P61970, P55786, P61247, P53396, P18669, Q13155, P17987, P49207, P39019, O75821, P50570, Q93008, P60842, P52306, P11940, P08238, P09211, P08237, P61254, Q15008, P63244, P56537, P61966, P34932, Q14974, Q02750, P54136, O94979, Q99832, P62195, P12814, P30041, P19105, O95336, P22102, P08243, Q7L576, P17980, P28482, P62906, Q00610, P50991, O95433, Q9Y617, O15144, P05388, P19525, P49327, Q14152, P31939, P07195, Q9UN86, Q9Y262, Q16658, Q9H9S4, P40429, P41091, Q92598, P63151, P55060, P47897, P06733, P62277, Q99460, P06737, P68371, P11586, P32969, P62280, P18621, Q5VT79, P68366, Q9NV70, Q13618, Q02543, Q92973, P78371, Q8WUM4, P22314, P62910, P62913, P06744, P35579, O76094, P35580, P21980, P23919, P52209, P61923, Q9P2J5, P62191, P35268, O95373, P61160, P13929, P27708, P09960, P60866, O43684, P60660, Q7Z6Z7, P55884, O60763, Q14204, Q9UNM6, Q14203, O14980, O00231, O00232 | 1.21E-100 |
| CC | GO:0070062 | | extracellular exosome | P46940, P07437, Q96QK1, P36578, Q01082, P04406, Q13200, P78417, O00571, Q9BR76, P43034, O43175, P23526, Q9NQW7, P07737, P53621, P13645, P61970, P55786, P62269, P61247, P53396, O43707, P62263, P23396, P62851, Q04828, P18669, P49588, P49207, P17987, P39019, P41252, P50570, O60701, O75369, Q562R1, P60842, P52306, P28074, Q13347, P11940, O00299, P08238, P08237, P09211, P00558, P61254, Q15008, P63244, P13796, P56537, P61966, Q8NC51, P40227, P34932, Q01813, Q14974, P62837, Q02750, Q9NZN4, P54136, P16152, Q99832, P62195, Q9H223, P12814, P30041, P62249, Q9NQH7, P19105, P00491, P22102, O95336, Q7L576, O75083, P28482, P62906, P62241, Q00610, O95433, P11216, P50991, P62244, P07947, P63104, Q5VTE0, Q9Y617, O15144, P05388, P00338, P83731, P49327, P61088, P31939, P07195, Q13085, Q16658, P35908, P14618, P50914, Q9H9S4, Q92598, P41091, P60228, O43242, P55060, P06733, P30153, P60709, P62277, P14174, Q99460, P61353, P06737, P11586, P29401, Q9H4M9, P68371, P62280, Q5VT79, P13489, P68366, O76013, Q9UQ80, Q13618, P04264, Q9BUF5, P15880, P40925, Q92973, P78371, Q8WUM4, P22314, P21333, Q04446, P62913, P06744, Q96FW1, Q07960, P60981, P35579, P35580, P21980, P52209, P11413, O95747, P51665, P56192, Q96TA1, Q8NBZ7, P14868, P35268, P46779, P61981, Q86VP6, P07900, P61160, Q12965, P13929, P27708, P09960, P46782, P46781, P60866, Q01518, P02765, P60660, P26639, P62081, Q7Z6Z7, Q9NTK5, P55884, Q04941, P49368, P13639, Q14204, Q9UNM6, Q9Y2A7, Q13885, P62333, P18077, P16070, O00231, O00232 | 1.29E-78 |
| CC | GO:0016020 | | membrane | Q13200, P04406, P36578, Q7L1Q6, O00203, Q12904, P62753, P27635, P07737, P53621, P13645, P52789, P62269, P53396, P62263, P23396, P18669, P49588, Q13155, P39019, P41252, Q93008, P60842, P11940, O00299, P08238, P00558, P61254, Q9NSD9, O00410, P61966, Q8NC51, P43686, Q01813, Q14974, P54136, P62195, P61221, P30041, Q9H223, P62249, P48147, P84098, P17980, P62906, P62241, Q00610, P11216, P62244, Q92616, P05388, P00338, P19525, P49327, Q14152, P83731, P31939, P07195, Q9Y262, P07814, P35908, P62841, P50914, P40429, P26373, P62847, P53618, P60228, O43242, P55060, P06733, P30153, P60709, P62277, Q9Y6E2, Q99460, P61353, Q9H4M9, P11586, P32969, P62280, Q9NV70, Q9UQ80, Q13618, P04264, O00303, O43747, P15880, Q02543, Q8WUM4, P21333, P62910, P06744, P62913, P35579, Q96S52, Q9BXJ9, Q13418, P11413, P51665, P62191, P56192, P46776, P14868, P46778, P46779, P61981, Q86VP6, O95373, P07900, P61160, Q7Z417, P27708, O00429, P46782, P46781, P60866, P60660, P62081, Q7Z6Z7, P25398, Q9NTK5, Q04941, O60763, P13639, Q9UNM6, Q14204, Q14203, O14980, P62333, P18077, O00231, O00232 | 1.40E-46 |
| CC | GO:0005737 | | cytoplasm | Q5SZL2, P46940, P07437, P36578, Q01082, P04406, Q7L1Q6, Q12904, Q9ULV4, P78417, Q9BR76, O00571, P62753, P23526, Q9NQW7, P14324, P07737, P53621, Q8NB66, P13645, P61247, Q9BQE3, P53396, O43707, P62263, P23396, P62851, Q08J23, P18669, P49588, P49207, P17987, P39019, O75821, P41252, P50570, P54577, O75369, Q93008, Q562R1, P20042, P60842, P11940, O00299, P08238, P08237, P09211, Q9NSD9, Q16831, P63244, Q9BUK6, P13797, P13796, P09497, P56537, O00410, Q8NC51, P43686, P40227, Q01813, Q14974, Q02750, P54136, O94979, Q99832, P62195, P61221, P12814, P30041, P00491, P22102, P48147, P84098, O75083, P28482, P17980, P62906, P62241, O95433, P11216, P50991, P62244, P07947, P63104, Q5VTE0, Q9Y617, O15144, P05388, Q92616, P19525, P00338, P83731, P49327, P61088, Q14152, P07195, Q9UN86, Q13085, P07814, Q16658, P35908, P62841, P14618, P50914, O43592, P40429, P62847, Q92598, P41091, P53618, P60228, Q13045, O43242, O60443, P55060, Q8NB90, P47897, P06733, P60709, Q9Y6E2, P14174, P06737, P32969, P62280, P13489, Q9NV70, Q9Y3I0, Q9UQ80, P10155, O43747, Q9BUF5, P15880, P40925, Q92973, P78371, Q8WUM4, P22314, P21333, P62913, P06744, Q96FW1, Q07960, P35579, O76094, P35580, O60841, Q9BY44, Q9BXJ9, Q13418, Q9P2J5, P11413, O95747, P62191, P56192, Q96TA1, P14868, P35268, P46778, P46779, Q14694, Q86VP6, O95373, P07900, P61160, Q7Z417, Q12965, Q9NPQ8, P13929, P09960, O00429, P46781, P60866, O75444, P26639, P62081, Q7Z6Z7, Q9NTK5, P55884, Q9H853, P49368, P13639, Q14203, O14980, Q13885, P16070, O00232 | 2.55E-36 |
| CC | GO:0005840 | | ribosome | P36578, P46776, P35268, P46778, P46779, P62277, P62753, P27635, P61353, P32969, P62280, P62249, P62269, P84098, P61247, P62906, P62241, P62244, P62263, P23396, P62851, P46781, P60866, P15880, Q02543, P49207, P39019, P05388, Q92616, P19525, P83731, P62081, P62910, P62913, P62841, P50914, P18077, P26373, P40429, P62847 | 1.15E-34 |
| MF | GO:0044822 | | poly(A) RNA binding | Q01082, P36578, Q7L1Q6, O60610, O00571, P62753, P27635, P14324, P07737, P62269, P61247, O43707, P23396, P62263, P62851, Q08J23, P17987, O75821, P39019, P54577, O75369, P60842, P20042, P11940, P08238, P61254, P63244, Q8NC51, O00410, P40227, Q14974, P62249, P84098, P62906, P62241, Q00610, P50991, P62244, P63104, Q92616, P05388, P19525, Q14152, P49327, P61088, P83731, Q9UN86, Q9Y262, Q16658, P62841, P50914, P14618, P26373, P40429, P62847, P60228, P06733, P62277, P61353, P62280, P18621, Q9Y3I0, Q9UQ80, P15880, Q02543, Q92973, P22314, P21333, P62910, P62913, P35579, O76094, O60841, Q9BXJ9, P62191, P46776, P14868, P46778, P35268, P46779, Q14694, P61981, P07900, Q7Z417, P09960, P46782, P46781, P60866, P62081, P25398, Q7Z6Z7, P49368, O60763, P13639, Q14204, P18077, Q99613 | 1.68E-42 |
| MF | GO:0003735 | | structural constituent of ribosome | P36578, P46776, P35268, P46778, P46779, P62277, P62753, P27635, P61353, P32969, P62280, P62249, P18621, P62269, P84098, P61247, P62906, P62241, P62244, P62263, P23396, P46782, P46781, P60866, P15880, Q02543, P49207, P39019, P05388, P83731, P62081, P62910, P62913, P25398, P61254, P62841, P50914, P18077, P26373, P40429, P62847 | 1.21E-29 |
| MF | GO:0098641 | | cadherin binding involved in cell-cell adhesion | P60228, P46940, Q96TA1, Q01082, Q01813, Q7L1Q6, P06733, Q9BR76, O00571, Q9Y6E2, P54136, Q9H4M9, P30041, Q9H223, P07737, O95433, P63104, P15880, P49207, Q92616, P00338, P83731, P49327, P21333, P31939, Q07960, Q9NTK5, O75369, P35579, O60763, O00299, P08238, P13639, Q16658, Q9BY44, P63244, P14618, P50914, Q8NC51, P41091 | 5.11E-24 |
| MF | GO:0005515 | | protein binding | P46940, P07437, Q13200, P36578, O60610, P78417, Q9BR76, P27635, P43034, P62753, P23526, P07737, P52789, P62269, Q9BQE3, P62263, P23396, P62851, Q04828, P41252, P54577, O75369, Q562R1, P20042, P28074, Q13347, P00558, Q9NSD9, P09497, O00410, Q8NC51, P43686, P40227, P62837, P35606, Q9NZN4, P62249, Q9Y678, P84098, P11216, P62244, P07947, P63104, P00338, P61088, P83731, Q13085, Q9BZF3, P07814, P62841, P50914, P14618, P26373, P53618, P60228, O43242, P30153, P60709, P29401, Q9H4M9, P13489, P04264, O00303, O43747, P15880, P40925, P52926, P21333, Q96FW1, Q07960, O60841, Q9BXJ9, Q13418, P11413, O95747, Q10567, P51665, P46776, P14868, P46778, P46779, Q14694, P61981, Q86VP6, P07900, Q8TF05, Q12965, Q9NPQ8, O00429, P46782, Q16401, P46781, O75444, P26639, P62081, P17096, Q9NTK5, P26640, P49368, P13639, Q9Y2A7, P62333, P18077, Q99613, Q96QK1, P04406, Q01082, Q12904, Q9ULV4, O00571, Q9Y6Q5, P61970, P61247, P53396, O43707, P18669, Q13155, P17987, P39019, O75821, P50570, Q93008, P60842, P52306, P11940, O00299, P08238, P09211, P08237, P61254, Q15008, P63244, P56537, P34932, Q14974, Q02750, P54136, O94979, Q99832, P62195, P61221, P12814, Q9H223, P30041, P19105, O95336, P08243, P48147, Q7L576, P28482, P17980, P62906, Q00610, P50991, O95433, O15144, P05388, P19525, P49327, Q14152, P07195, Q9UN86, Q9Y262, Q16658, P35908, P18887, Q9H9S4, P41091, Q92598, Q13045, P63151, P55060, P47897, P06733, P62277, P14174, P06737, P11586, P32969, P62280, P18621, P68366, Q9NV70, Q9Y3I0, Q9UQ80, Q13618, Q99062, Q9BYX2, Q02543, Q92973, P78371, Q8WUM4, P22314, P62913, P60981, P35579, Q96S52, O76094, P35580, P21980, Q9BY44, Q9P2J5, P62191, P35268, O95373, Q7Z417, P45974, P09960, P60866, O43684, P60660, P52888, Q7Z6Z7, P55884, Q04941, O60763, Q14204, Q14203, O14980, Q9H993, Q13885, P16070, O00231 | 1.30E-19 |
| MF | GO:0003723 | | RNA binding | P36578, P46776, P35268, P46778, P46779, P47897, O00571, P32969, P62249, Q7Z417, Q86TM3, P62269, P84098, P61247, P62906, P10155, P62244, P62263, P23396, P46782, P62851, P60866, P15880, Q02543, P49207, O75821, P26639, P83731, P62081, Q14152, P62913, P55884, P20042, P11940, P61254, O14980, Q9NSD9, P62841, P50914, P26373 | 2.65E-14 |
| **Down-regulated proteins at 24 hpi** | | | | | |
| BP | GO:0000398 | | mRNA splicing, via spliceosome | P22626, Q15029, P14678, Q15717, Q13435, P62314, P62316, P08621, Q15393, Q6P2Q9, Q14103, P38919, P09651, Q01130, P09661 | 3.91E-12 |
| BP | GO:0008380 | | RNA splicing | Q9Y2W2, Q15029, P23246, P14678, Q13435, P62314, P62316, Q15393, P08621, Q6P2Q9, P38919, P09661, Q01130 | 3.08E-11 |
| BP | GO:0006397 | | mRNA processing | Q13435, P22626, P08621, Q15029, Q15393, Q6P2Q9, P23246, Q9UKA9, P09651, Q01130 | 2.43E-07 |
| BP | GO:0051170 | | nuclear import | P62314, P62316, P09651, P14678 | 9.01E-05 |
| BP | GO:0006396 | | RNA processing | O14979, P62314, O15042, Q9Y2W2, Q14103, Q8IWX8 | 1.18E-04 |
| CC | GO:0005681 | | spliceosomal complex | O14979, P22626, Q9Y2W2, Q15029, P14678, Q13435, P62316, P08621, Q15393, Q6P2Q9, Q9UKA9, P09651, Q01130, P09661 | 4.84E-16 |
| CC | GO:0071013 | | catalytic step 2 spliceosome | Q13435, P62314, P62316, P22626, Q15029, Q15393, Q6P2Q9, P38919, P09651, P09661, P14678 | 1.68E-11 |
| CC | GO:0019013 | | viral nucleocapsid | O14979, P62314, P08621, Q15029, Q14103, P09651, P09661, P14678 | 4.44E-11 |
| CC | GO:0030529 | | intracellular ribonucleoprotein complex | O14979, P62314, P22626, Q15029, Q14103, P38919, P09651, P09661, P14678 | 2.54E-07 |
| CC | GO:0005654 | | nucleoplasm | Q92925, Q9Y3Y2, Q9Y2W2, Q01970, Q15029, P23246, P78527, P14678, Q15717, Q16666, Q6P2Q9, P38919, Q99575, P09651, O14979, Q8N2I9, Q13835, P22626, Q08752, Q93009, Q9UHR4, Q13435, P62314, P62316, O15042, Q15393, P08621, P78346, Q14103, P43243, Q01130, P09661, P29590 | 5.23E-07 |
| MF | GO:0044822 | | poly(A) RNA binding | Q9Y3Y2, Q9Y2W2, Q15029, P23246, O95147, P78527, P14678, Q15717, Q86Y79, Q16666, Q6P2Q9, Q86SE5, Q9UKA9, P38919, Q99575, P09651, O14979, P22626, Q00059, Q8IWX8, Q13435, P62314, P62316, O15042, Q969Z0, P08621, P78346, Q14103, P43243, Q01130, P09661 | 6.41E-15 |
| MF | GO:0005515 | | protein binding | P01130, Q92925, P21926, P36405, Q9Y3Y2, Q15029, P23246, P14678, P26006, P11166, Q15717, Q86Y79, P49458, P05121, Q16666, Q86SE5, P09651, P22626, Q00059, P35354, P30533, Q8WV24, P04035, Q8IWX8, Q93009, Q08752, Q9UHR4, Q9UKV5, Q8TED9, P62314, P62316, Q969Z0, P10909, Q15393, P09661, O14763, Q5VU43, Q9NVA2, Q9Y2W2, Q86VP1, O95147, P78527, Q9BY76, Q9P2B2, P01008, P18065, Q6P2Q9, P38919, Q99575, O14979, Q13835, Q8N2I9, Q9UJW8, P14923, P05067, Q9BT40, P20337, Q13435, P53801, O15042, P08621, P78346, Q14103, P43243, Q14249, Q01130, P29590, P02794 | 1.21E-07 |
| MF | GO:0000166 | | nucleotide binding | O14979, Q15717, O15042, P22626, P08621, Q14103, P43243, Q86SE5, P23246, Q9UKA9, P09651, Q01130 | 1.18E-06 |
| MF | GO:0003723 | | RNA binding | P22626, Q8IWX8, P14678, Q15717, P49458, P62314, O15042, P08621, Q15393, P78346, Q6P2Q9, Q14103, Q9UKA9, P09651 | 2.87E-06 |
| MF | GO:0003676 | | nucleic acid binding | O14979, Q9UJW8, P22626, P23246, O15042, P08621, Q15393, Q14103, P43243, P38919, Q9UKA9, Q86SE5, Q14249, P09651, Q01130 | 3.34E-04 |
| **Up-regulated proteins at 24 hpi** | | | | | |
| BP | GO:0006614 | | SRP-dependent cotranslational protein targeting to membrane | P15880, Q02543, P05387, P46778, P62910, P62913, P25398, P62280, P62979, P62899, P18621, P61254, P61247, P62906, P47914, P18077, P40429, P46781 | 4.75E-19 |
| BP | GO:0006413 | | translational initiation | P15880, Q02543, P05387, P46778, P62910, P62913, P25398, P62280, Q15056, P62979, P11940, P62899, P18621, P61254, P61247, P62906, P47914, P18077, P40429, P46781 | 6.75E-19 |
| BP | GO:0000184 | | nuclear-transcribed mRNA catabolic process, nonsense-mediated decay | P15880, Q02543, P05387, P46778, P62910, P62913, P25398, P62280, P62979, P11940, P62899, P18621, P61254, P61247, P62906, P47914, P18077, P40429, P46781 | 1.19E-18 |
| BP | GO:0019083 | | viral transcription | P15880, Q02543, P05387, P46778, P62910, P62913, P25398, P62280, P62979, P62899, P18621, P61254, P61247, P62906, P47914, P18077, P40429, P46781 | 1.07E-17 |
| BP | GO:0098609 | | cell-cell adhesion | P15880, Q01813, P06733, P04075, P31947, P00338, P29692, P31939, P30041, P07737, Q15056, Q05682, P13639, Q16658, P31949, P0DMV8, P63244, P47914, P14618, Q9BZ29, Q99497, Q8NC51, P63104 | 1.14E-16 |
| CC | GO:0070062 | | extracellular exosome | Q96QK1, P07437, P04406, P48739, P78417, P31947, P06733, P12268, P27816, P14174, P23528, O43175, P68371, P29401, P62280, P07737, P62899, P62993, P68366, P31949, P61247, P53396, P08758, Q04828, Q99497, P15880, P06702, P22528, O14910, P04083, P18669, Q9HD42, P06703, O60884, P05109, P62913, P35579, Q92734, P11940, O00299, P21980, P26447, P00558, P09525, P09211, P61254, P10599, P63244, Q8NC51, Q01813, P25787, P00441, P52565, P07476, P04075, O75436, P30041, P62979, Q9NQH7, P19105, P63208, P50995, P09960, P29966, P62906, P07947, P63104, P46781, Q5VTE0, Q9H299, P60174, P05387, P00338, P31939, P07195, P62937, P13639, P12035, Q16658, Q13885, P14618, P18077, P20073 | 1.86E-35 |
| CC | GO:0005829 | | cytosol | Q96QK1, P04406, P78417, Q4KMP7, P31947, P06733, P29692, P12268, O43175, Q4VCS5, P68371, P29401, P62280, P07737, P62899, P18621, P62993, P68366, P61247, P53396, P47914, Q4G0F5, Q5TB80, Q04828, Q99497, P15880, P06702, Q02543, P18669, P06703, P62910, O60884, P05109, P54577, P62913, Q8WV28, P35579, Q92734, P11940, P21980, Q05682, P00558, P09211, Q92529, P61254, P10599, P63244, P24534, Q01813, P25787, P00441, P46778, P52565, P04075, O75436, P30041, P62979, Q02224, Q15056, P19105, Q15058, P08243, P63208, P09960, P62906, P07947, Q9BZ29, P63104, P46781, P60174, P05387, P00338, P31939, P19838, P25398, P07195, P62937, P13639, Q16658, P0DMV8, P14618, P18077, P20073, P40429, P02795 | 5.12E-32 |
| CC | GO:0005913 | | cell-cell adherens junction | Q01813, P06733, P31947, P04075, P29692, P30041, P07737, Q15056, P31949, P47914, Q99497, Q9BZ29, P63104, P15880, P04083, P00338, P31939, P35579, O00299, Q05682, P13639, Q16658, P0DMV8, P63244, P14618, Q8NC51 | 3.57E-19 |
| CC | GO:0022625 | | cytosolic large ribosomal subunit | Q02543, P18621, P62899, P05387, P61254, P46778, P62906, P62910, P47914, P62913, P18077, P40429 | 1.58E-12 |
| CC | GO:0005737 | | cytoplasm | P07437, P04406, P52597, Q8NB90, P78417, P31947, P06733, P29692, P12268, P27816, P14174, P23528, Q4VCS5, P62280, P07737, P62993, P31949, P61247, Q9BQE3, P53396, P08758, Q8IY21, Q5TB80, Q99497, P15880, P22528, P04083, P18669, P06703, P54577, P62913, Q8WV28, P35579, Q92734, P11940, O00299, P09525, P09211, P10599, P63244, P24534, Q8NC51, P57058, Q01813, P25787, P00441, P46778, P52565, P07476, O75534, Q9Y250, P30041, P62979, Q02224, P63208, P50995, P09960, P51858, P62906, P07947, P63104, P46781, Q5VTE0, Q9H299, O15347, P00338, P07195, P19838, Q9H853, P13639, Q16658, P0DMV8, Q13885, P14618, P40429, P02795 | 2.34E-12 |
| MF | GO:0098641 | | cadherin binding involved in cell-cell adhesion | Q01813, P06733, P31947, P04075, P29692, P30041, P07737, Q15056, P31949, P47914, Q99497, Q9BZ29, P63104, P15880, P04083, P00338, P31939, P35579, O00299, Q05682, P13639, Q16658, P0DMV8, P63244, P14618, Q8NC51 | 2.33E-19 |
| MF | GO:0044822 | | poly(A) RNA binding | P52597, P46778, P04075, P06733, P31948, O75534, P27816, P62280, P62979, P07737, Q15056, P18621, P62899, P62993, P50995, P09960, P62906, P51858, P61247, P47914, P46781, P63104, P15880, O15347, Q02543, P62910, P62913, P54577, P25398, P35579, P62937, P11940, P26447, P13639, Q16658, P61254, P10599, P63244, P14618, P18077, P20073, P40429, Q8NC51 | 2.12E-18 |
| MF | GO:0003735 | | structural constituent of ribosome | P15880, Q02543, P05387, P46778, P62910, P62913, P25398, P62280, P62979, P62899, P18621, P61254, P61247, P62906, P47914, P18077, P40429, P46781 | 1.42E-12 |
| MF | GO:0005515 | | protein binding | Q96QK1, P07437, P04406, P52597, Q4KMP7, P78417, P29692, P27816, P12268, P23528, Q4VCS5, P07737, Q9BQE3, P61247, P53396, P08758, Q8IY21, Q04828, Q99497, P06702, O14910, P04083, P18669, P06703, O60884, P54577, P50479, Q8WV28, Q92734, Q9NVC3, P11940, O00299, P00558, P09211, P10599, P61254, Q92529, P63244, Q8NC51, P52565, P04075, Q9Y250, P30041, P62979, Q02224, P19105, P08243, P50995, P63208, P62906, P07947, Q9BZ29, P63104, P27694, P60174, O15347, P05387, P00338, P07195, Q96DT7, Q16658, P14618, P29034, P02795, P31947, P06733, P31948, P14174, P29401, P62280, P62899, P18621, P62993, P68366, P31949, Q5TB80, Q9BYX2, P15880, Q02543, Q9HD42, P05109, P62913, P35579, P21980, P26447, Q05682, P09525, P24534, P25787, P00441, P46778, P07476, O75534, O75436, Q15056, Q8TF05, Q15058, P09960, P46781, P19838, P62937, P13639, P0DMV8, Q13885, P18077, P20073 | 1.86E-11 |
| MF | GO:0048306 | | calcium-dependent protein binding | P04083, P26447, P09525, P06703, P50995, P31949, P20073 | 5.66E-06 |

BP: biological process; CC: cellular component; MF: molecular function
