# Supplementary material for: Dataset supporting the proteomic characterization of human corneal epithelial cells with HSV-1 infection
Source: Data Brief. 2019 Sep 28;27:104579. doi: 10.1016/j.dib.2019.104579 (PMC6817629; doi:10.1016/j.dib.2019.104579)
Supplement: Multimedia component 4 [file mmc4.doc]

Supplementary table 2. KEGG pathways associated with the dysregulated proteins in HCECs in response to HSV-1 infection at 6 and 24 hpi.

| **KEGG pathway ID** | **Terms** | **Proteins involved** | **P value** |
| --- | --- | --- | --- |
| **Down-regulated proteins at 6 hpi** | | | |
| hsa03040 | Spliceosome | Q12874, P26368, P09234, Q8IWX8, Q9BWJ5, P62304, Q07955, Q13435, P55769, P62316, Q9UHX1, Q16629, O75400, P08621, Q15393, Q15459, Q01130, P09661 | 2.98E-12 |
| hsa00020 | Citrate cycle (TCA cycle) | P31040, P40926, P50213, P07954, P36957, P09622, P08559, Q9P2R7 | 1.45E-07 |
| hsa04141 | Protein processing in endoplasmic reticulum | P30101, Q8NBS9, P27797, Q99442, P30040, P13667, P14314, Q9NYU2, Q14697, Q96HE7, P07237, P11021, Q9Y4L1 | 4.06E-06 |
| hsa01130 | Biosynthesis of antibiotics | P40926, O76062, P09110, P09622, P31040, P54819, P30084, P07954, P50213, P08559, P36957, Q9P2R7, P49419 | 4.05E-05 |
| hsa01200 | Carbon metabolism | P31040, P40926, P30084, P50213, P07954, P36957, P09622, P08559, Q9P2R7 | 1.85E-04 |
| hsa00620 | Pyruvate metabolism | P40926, P07954, P09622, P08559, P49419 | 0.002351 |
| hsa00071 | Fatty acid degradation | P49748, P30084, P09110, P49419, Q15067 | 0.003344 |
| hsa05012 | Parkinson's disease | P99999, P19404, P31040, P06576, P14854, P30049, P30405, O43678 | 0.003892 |
| hsa01100 | Metabolic pathways | P49748, O15460, O76062, P07686, O43678, P09622, P31040, P54819, P22307, Q4KWH8, P30049, P08559, P36957, P49419, P19404, P40926, P14854, P09110, P99999, P06576, Q13510, P12532, Q14697, P30084, P50213, P07954, O00330, Q9P2R7, Q15067 | 0.005396 |
| hsa05016 | Huntington's disease | P99999, P19404, P31040, P06576, P14854, P04179, P30049, P30405, O43678 | 0.005713 |
| **Up-regulated proteins at 6 hpi** | | | |
| hsa03010 | Ribosome | P36578, P46776, P35268, P46778, P46779, P62277, P62753, P27635, P61353, P32969, P62280, P62249, P18621, P62269, P84098, P61247, P62906, P62241, P62244, P62263, P23396, P46782, P62851, P46781, P60866, P15880, Q02543, P49207, P39019, P05388, P83731, P62081, P62910, P62913, P25398, P61254, P62841, P50914, P18077, P40429, P26373, P62847 | 4.59E-31 |
| hsa03050 | Proteasome | P43686, O43242, Q13200, Q99460, P62195, P28074, Q9UNM6, Q15008, P17980, P62333, P51665, O00231, P62191, O00232 | 2.32E-10 |
| hsa01130 | Biosynthesis of antibiotics | P40925, P04406, Q01813, Q9Y617, P18669, P06733, P00338, P06744, P07195, P31939, O43175, P29401, P14324, Q15738, P22102, O95336, P13929, P08237, P00558, P52789, P52209, P11413, P53396, P14618 | 6.22E-08 |
| hsa01200 | Carbon metabolism | P40925, P04406, Q9Y617, Q01813, P18669, P06733, P06744, O43175, P29401, O95336, P13929, P00558, P08237, P52789, P52209, P11413, P14618 | 1.88E-07 |
| hsa00970 | Aminoacyl-tRNA biosynthesis | P26640, P56192, P07814, P14868, P49588, Q9P2J5, P26639, P47897, Q9NSD9, P41252, P54577, P54136 | 3.23E-06 |
| hsa00010 | Glycolysis / Gluconeogenesis | P04406, Q01813, P13929, P18669, P00558, P08237, P52789, P00338, P06733, P14618, P06744, P07195 | 3.76E-06 |
| hsa05169 | Epstein-Barr virus infection | P43686, O43242, Q13200, P19525, Q99460, P61981, P62195, Q9UNM6, O14980, Q15008, P17980, P51665, P62333, O00231, P16070, P62191, O00232, P63104 | 4.66E-05 |
| hsa01230 | Biosynthesis of amino acids | P04406, Q9Y617, Q01813, P13929, P18669, P00558, P08237, P06733, P14618, O43175, P29401 | 6.05E-05 |
| hsa00030 | Pentose phosphate pathway | Q01813, O95336, P08237, P11413, P52209, P06744, P29401 | 1.75E-04 |
| hsa03013 | RNA transport | P60228, Q14974, O75821, Q14152, P55884, P20042, P60842, Q13347, P11940, O60841, O14980, Q7L576, O43592, O00303, Q99613, P41091 | 1.75E-04 |
| **Down-regulated proteins at 24 hpi** | | | |
| hsa03040 | Spliceosome | Q9Y2W2, Q15029, Q8IWX8, P14678, Q13435, P62314, P62316, O15042, P08621, Q15393, Q6P2Q9, P38919, P09651, P09661, Q01130 | 9.54E-14 |
| hsa04976 | Bile secretion | P11166, P01130, P04035 | 0.079716 |
| **Up-regulated proteins at 24 hpi** | | | |
| hsa03010 | Ribosome | P15880, Q02543, P05387, P46778, P62910, P62913, P25398, P62280, P62979, P62899, P18621, P61254, P61247, P62906, P47914, P18077, P40429, P46781 | 1.99E-13 |
| hsa00010 | Glycolysis / Gluconeogenesis | P60174, P04406, Q01813, P18669, P00558, P04075, P00338, P06733, P14618, P07195 | 7.30E-08 |
| hsa01230 | Biosynthesis of amino acids | P60174, P04406, Q01813, P18669, P00558, P04075, P06733, P14618, O43175, P29401 | 1.77E-07 |
| hsa01130 | Biosynthesis of antibiotics | P04406, P60174, Q01813, P18669, P06733, P00338, P04075, P07195, P31939, O43175, P29401, P00558, P53396, P14618 | 1.02E-06 |
| hsa01200 | Carbon metabolism | P60174, P04406, Q01813, P18669, P00558, P04075, P06733, P14618, O43175, P29401 | 6.56E-06 |
| hsa05130 | Pathogenic Escherichia coli infection | P07437, P68366, Q9BQE3, Q13885, P68371 | 0.003014 |
| hsa04540 | Gap junction | P07437, P62993, P68366, Q9BQE3, Q13885, P68371 | 0.003776 |
| hsa00030 | Pentose phosphate pathway | Q01813, P04075, P29401 | 0.04593 |
| hsa00051 | Fructose and mannose metabolism | P60174, Q01813, P04075 | 0.054854 |
| hsa00620 | Pyruvate metabolism | P00338, P14618, P07195 | 0.081227 |
